# Accelerated Oxalosis Contributing to Delayed Graft Function after Renal Transplantation

**DOI:** 10.1155/2019/8942062

**Published:** 2019-03-25

**Authors:** Yvelynne P. Kelly, Astrid Weins, Melissa Y. Yeung

**Affiliations:** ^1^Department of Nephrology, Brigham and Women's Hospital, 75 Francis Street, Boston, MA 02115, USA; ^2^Department of Histopathology, Brigham and Women's Hospital, 75 Francis Street, Boston, MA 02115, USA

## Abstract

Hyperoxaluria is an important and underrecognized cause for allograft dysfunction and loss after transplantation. It is potentially treatable if recognized in a timely fashion. Research is ongoing to expand the array of therapeutic options available to treat this. We present a case of a 59-year-old gentleman who underwent deceased donor renal transplantation that was complicated by delayed graft function necessitating continuation of renal replacement therapy. His initial biopsy showed extensive acute tubular necrosis with associated peritubular capillaritis and interstitial nephritis and oxalate crystals in several tubules. Despite receiving methylprednisolone to treat moderate acute cellular rejection, he remained dialysis dependent with minimal urine output. An interval renal allograft biopsy revealed residual acute tubular necrosis with extensive oxalate crystals now visible in many tubules. His plasma oxalate level was concurrently elevated to 19.3 *μ*mol/L (reference range ≤ 1.9 *μ*mol/L). He commenced calcium citrate to manage his hyperoxaluria and ultimately became dialysis independent at 3 weeks after transplantation. This case provides an important example of accelerated oxalate nephropathy as an underappreciated contributor to delayed graft function after renal transplantation. Our accompanying discussion provides an update on current therapeutic measures for managing this challenging condition.

## 1. Case Report

A 59-year-old gentleman with end-stage kidney disease due to hepatitis C with focal segmental glomerulosclerosis, on hemodialysis for seven years, underwent deceased donor renal transplantation. The donor kidney had a KDPI of 60%. There was a 4 antigen HLA mismatch with Class II panel reactive assay (PRA) of 62.31%. Class I PRA was 0%. His donor specific antibody testing was positive for an antibody to DQ7. The donor had died as a result of anoxic brain injury following cardiopulmonary arrest resulting from anaphylactic shock. The cold ischemic time was 12 hours, 7 minutes and the warm ischemic time was 51 minutes. Basilixumab was used for immunosuppression induction and the patient underwent early steroid withdrawal. His maintenance immunosuppression was tacrolimus (targeting trough levels 5-8) and mycophenolate sodium 720 mg BID.

**Figure 1:**
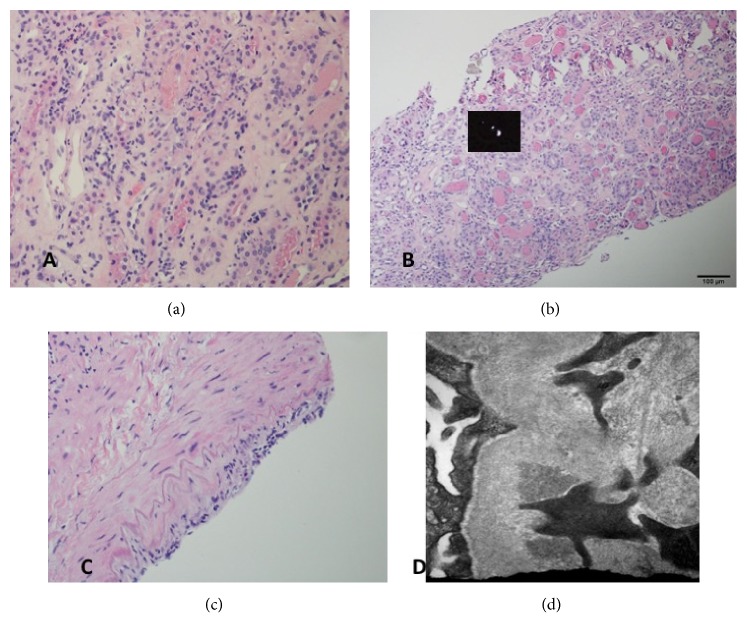
(a) H&E stain showing extensive acute tubular necrosis with associated peritubular capillaritis and interstitial nephritis; (b) H&E stain showing polarizable oxalate crystals in a tubule; (c) H&E stain showing active endothelialitis in one large caliber artery, (d) electron microscopy showing electron-dense deposits with immunoreactivity for IgA, in keeping with a remote IgA-dominant immune complex mediated nephropathy in the donor.

The patient's postoperative course was complicated by delayed graft function, necessitating continuation of renal replacement therapy. He was hemodynamically stable throughout his hospital course. A renal allograft ultrasound showed good blood flow to the entire kidney with a resistive index of 0.64. A repeat flow crossmatch was negative, but he remained positive for a persistent low-level donor specific antibody to DQ7 (1000 MFI). He underwent a renal transplant biopsy on his 4th day postoperatively. This showed extensive acute tubular necrosis with associated peritubular capillaritis and interstitial nephritis (Figure 1). Oxalate crystals were seen in several tubules. One large caliber artery showed active endothelialitis, but no tubulitis or glomerulitis seen. C4d staining was negative in the peritubular capillaries. Electron microscopy revealed minimal effacement of podocyte foot processes. The patient received methylprednisolone 500 mg x 3 doses to treat moderate acute cellular rejection. His tacrolimus dose was also optimized as his trough levels had been running low at between 3.5 and 6. He continued to take mycophenolate sodium at a dose of 720 mg BID. A decision was made not to treat for antibody-mediated rejection given that no glomerulitis was seen and that there was minimal capillaritis with a negative C4d stain.

**Figure 2:**
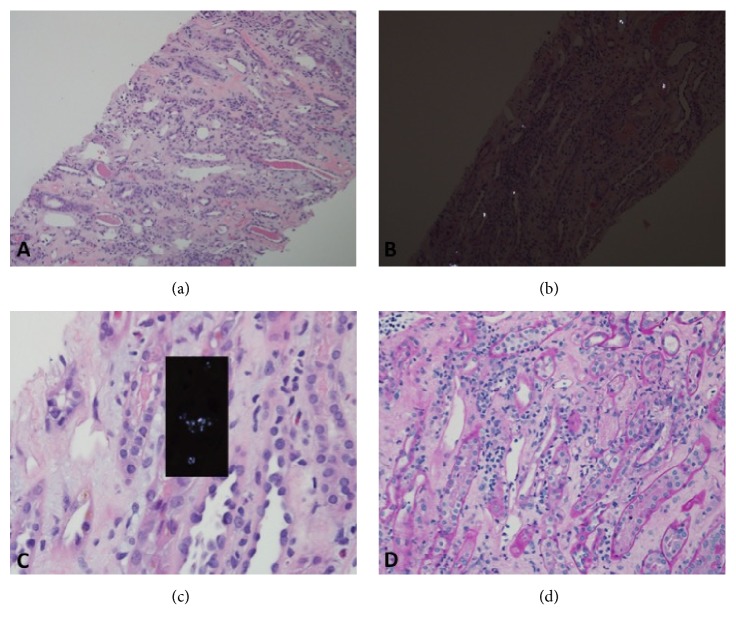
(a) H+E stain revealing residual acute tubular injury with focal epithelial necrosis and associated mild peritubular capillaritis and interstitial nephritis; (b) H+E stain showing polarizable oxalate crystals in several tubules; (c) H&E stain revealing acute tubular necrosis and oxalate crystals in the tubules; (d) PAS stain showing dilated peritubular capillaries with capillaritis and active interstitial nephritis.

He remained dialysis dependent with minimal urine output for three weeks after transplantation. A urine protein : creatinine ratio was elevated to 1070 mg/g when assessed after hospital discharge when his urine output started to gradually improve. His urine microalbumin : creatinine ratio was 450 mg/g. A decision was ultimately made to readmit the patient on day 12 after transplantation for IV thymoglobulin 1.5 mg/kg to treat his Banff Type IIa T cell mediated rejection, given his lack of response to pulsed IV steroid treatment. Given lack of improvement in renal function and urine output by day 16 after transplantation, a second renal allograft biopsy was performed. This revealed residual acute tubular necrosis with associated mild peritubular capillaritis and interstitial nephritis (Figure 2). Extensive oxalate crystals were now visible in many tubules. His plasma oxalate level was concurrently elevated to 19.3 *μ*mol/L (reference range ≤ 1.9 *μ*mol/L). No signs of persistent acute antibody- or cell-mediated rejection were seen. Again, there was minimal segmental effacement of the podocyte foot processes seen on electron microscopy and no evidence of recurrent focal segmental glomerulosclerosis present.

He commenced calcium citrate along with dietary oxalate restriction to manage his hyperoxaluria and his serum creatinine improved to a nadir of 1 mg/dl (88 *μ*mol/L), with a concomitant gradual reduction in his proteinuria and microalbuminuria to undetectable levels. Of note, the patient had no history of malabsorptive intestinal disease and denied any GI symptoms throughout this time period. He had never suffered from renal calculi. At three weeks after transplantation, his urine output and creatinine clearance had recovered sufficiently to enable him to become dialysis independent. A decision was made to continue low-dose oral steroids in the long-term given the presence of early acute cellular rejection on his first allograft biopsy. An interval renal biopsy performed 2 months later showed no ongoing evidence of oxalate deposition, tubular necrosis or cellular rejection. A concurrent repeat plasma oxalate level showed interval reduction to the normal range.

## 2. Discussion

Hyperoxaluria is an important and underrecognized cause for allograft dysfunction and loss after transplantation. Oxalate is eliminated almost exclusively by the kidneys and is readily filtered at the glomerulus [1]. In states of acute or chronic hyperoxaluria, it can crystallize in the tubular lumen, injure the tubular epithelium, and obstruct the tubular lumen. It has also been linked to risk of recurrent urinary tract infections and the development of tubulointerstitial nephritis and ultimate interstitial fibrosis [2]. This leads to proteinuria due to the effects of acute tubular injury and, later, chronic tubulointerstitial damage [3].

After successful renal transplantation, excess plasma oxalate is cleared, resulting in transient hyperoxaluria lasting from 3 days to 3 weeks [4]. In some patients this process can be accelerated with dramatic clearance of plasma oxalate up to a year after transplantation, leading to delayed graft function and potentially poor long-term graft survival [5, 6]. Cases of oxalate nephropathy causing impaired renal function have also been described many years after renal transplantation in the setting of excessive dietary oxalate intake, including high doses of vitamin C [7, 8]. The presence of preexisting chronic allograft dysfunction can act as an additional risk factor for hyperoxaluria in this setting. Acute oxalate nephropathy has also been found to complicate nonrenal solid organ transplantation as well, leading to potential need for renal replacement therapy for the short and long term [9].

Our institution recently published a retrospective cohort study [10] of patients who had kidney allograft biopsies performed within three months of transplant here at Brigham and Women's Hospital, in order to examine the association between calcium oxalate deposition and the composite outcome of death or allograft failure within 5 years. 19.4% (67/346) of all allograft biopsies within three months of transplantation had calcium oxalate deposition. A multivariable logistic regression model revealed that higher serum creatinine, longer time on dialysis, and diabetes were independently associated with calcium oxalate deposition. Calcium oxalate deposition was strongly associated with delayed graft function and with increased hazard of the composite outcome when adjusted for black recipient race, donor type, time on dialysis before transplantation, diabetes and borderline, or acute rejection. Given the retrospective nature of this study, however, one cannot infer a causal association between oxalate deposition and allograft loss or death. Concurrent plasma oxalate measurements and information regarding dietary oxalate intake were not available for this retrospective cohort.

Recurrent primary hyperoxaluria has also led to early posttransplant allograft loss [11]. Combined liver-kidney transplantation is generally required in these cases as renal transplantation alone is unlikely to be successful due to persistent hyperoxaluria and nephrocalcinosis in the face of the ongoing enzyme deficiency at the hepatic level. In the pediatric literature [12], bilateral nephrectomy at the time of liver-kidney transplantation has been described to reduce total body oxalate load, along with continuation of hemodialysis postoperatively until predialysis serum oxalate levels fall < 20 pmol/L. For those with a very high total body oxalate load, sequential liver transplantation followed by kidney transplantation has also been advocated to prevent renal allograft loss [13].

Severe acute tubular necrosis can also promote formation of calcium oxalate crystals in renal tissue [14]. Progressive ATN may therefore have played a role in the severity of oxalate deposition in this case. Transplant rejection does not specifically increase oxalate deposition in the kidney, but the decreased glomerular filtration rate associated with moderate-severe transplant rejection can indirectly lead to increased oxalate crystal precipitation due to inability to filter oxalate through damaged, inflamed glomeruli.

Dietary intervention plays an important role in reducing the risk of recurrent hyperoxaluria after transplantation for those at risk of this [15]. A low oxalate diet (40-50 mg/day) has been advocated, along with use of cholestyramine, sodium bicarbonate, and calcium carbonate preoperatively. The role for probiotics to enhance* Oxalobacter formigenes* gut colonization to aid degradation and excretion of oxalate has not yet been fully determined. Trials to date have not shown significant reductions in urine or plasma oxalate concentrations, though the probiotic treatment has been well-tolerated and found to be successfully delivered to the gastrointestinal tract [16, 17]. An oral nonabsorbed enzyme designed to degrade both dietary and endogenously produced oxalate for severe hyperoxaluria has received orphan drug designation from the FDA and is under development at present [18, 19].

Intensification of the hemodialysis regimen is also utilized to manage recurrent hyperoxaluria after transplantation [15]. Daily 6-hour hemodialysis treatments are recommended for one week before transplantation, as well as after transplantation if the patient remains oliguric, along with an intensified oral fluid regimen postoperatively to aim for a urine output of > 2 L day. Loop diuretics should be avoided in the direct posttransplant period. Dialysis is particularly effective at removing oxalate, with approximately 75-90% reduction in plasma oxalate following one hemodialysis session, though with a small rebound of approximately 2 *μ*mol/L occurring within two hours after dialysis [20, 21].

For those who are not known to have been actively hyperoxaluric before transplantation, we would advocate checking either a plasma oxalate level or a 24-hour urine collection for oxalate clearance to assess for high plasma oxalate levels or hyperoxaluria preoperatively. A study of 212 patients [22] admitted for transplantation showed that plasma oxalate was on average 3 times above the normal limit in 98% of patients at the time of transplantation and remained elevated at 3 weeks after transplantation for 37% of patients. If a high plasma oxalate or hyperoxaluria is present, one could consider transiently intensifying the hemodialysis prescription before transplantation to lower total body oxalate as much as possible to reduce the risk of accelerated oxalate excretion and potential nephropathy associated with this after transplantation.

In summary, therefore, our case describes a renal allograft recipient, not previously noted to have high plasma or urine oxalate levels, who presents with accelerated oxalate nephropathy and hyperoxaluria after renal transplantation, contributing to delayed graft function and risk for allograft loss. Delayed oxalate excretion was likely worsened in the setting of extensive acute tubular necrosis related to prolonged cold ischemic time, as well as Banff Type IIa acute cellular rejection. However, greater recognition of the risk for accelerated oxalosis preoperatively will enable us to optimize protocols to lower total body oxalate where it is significantly elevated, in order to reduce the additional risk of delayed graft function posed by this.

## References

[1] Glew R., Sun Y., Horowitz B. L., Konstantinov K. N., Barry M., Fair et al. J. R. (2014). Nephropathy in dietary hyperoxaluria: a potentially preventable acute or chronic kidney disease. *World Journal of Nephrology*.

[2] Özdemir B. H., Ayva Ş., Özdemir G. (2018). Renal allograft with calcium oxalate deposition: association with urinary tract infection and development of interstitial fibrosis. *Experimental and Clinical Transplantation*.

[3] Cartery C., Faguer S., Karras A. (2011). Oxalate nephropathy associated with chronic pancreatitis. *Clinical Journal of the American Society of Nephrology*.

[4] Bagnasco S. M., Mohammed B. S., Mani H. (2009). Oxalate deposits in biopsies from native and transplanted kidneys, and impact on graft function. *Nephrology Dialysis Transplantation *.

[5] Truong L. D., Yakupoglu U., Feig D. (2004). Calcium oxalate deposition in renal allografts: morphologic spectrum and clinical implications. *American Journal of Transplantation*.

[6] Pinheiro H. S., Saraiva Câmara N. O., Osaki K. S., Ribeiro De Moura L. A., Pacheco-Silva A. (2005). Early presence of calcium oxalate deposition in kidney graft biopsies is associated with poor long-term graft survival. *American Journal of Transplantation*.

[7] Moyses-Neto M., Brito B. R. S., De Araújo Brito D. J. (2018). Vitamin C-induced oxalate nephropathy in a renal transplant patient related to excessive ingestion of cashew pseudofruit (Anacardium occidentale L.): a case report. *BMC Nephrology*.

[8] Suneja M., Kumar A. B. (2013). Secondary oxalosis induced acute kidney injury in allograft kidneys. *Clinical Kidney Journal*.

[9] Lefaucheur C., Hill G. S., Amrein C. (2006). Acute oxalate nephropathy: a new etiology for acute renal failure following nonrenal solid organ transplantation. *American Journal of Transplantation*.

[10] Palsson R., Chandraker A. K., Curhan G. C., Rennke H. G., McMahon G. M., Waikar S. S. (2018). The association of calcium oxalate deposition in kidney allografts with graft and patient survival. *Nephrology Dialysis Transplantation *.

[11] Liu S., Gao B., Wang G., Wang W., Lian X., Wu et al. S. (2018). Recurrent primary hyperoxaluria type 2 leads to early post transplant renal function loss: a case report. *Experimental and Therapeutic Medicine*.

[12] Lee E., Ramos-Gonzalez G., Rodig N., Elisofon S., Vakili K., Kim H. B. (2018). Bilateral native nephrectomy to reduce oxalate stores in children at the time of combined liver–kidney transplantation for primary hyperoxaluria type 1. *Pediatric Nephrology*.

[13] Alkunaizi A. M., Al-Sannaa N. A., Raslan W. F. (2012). Hyperoxaluria and rapid development of renal failure following a combined liver and kidney transplantation: emphasis on sequential transplantation. *Journal of Inherited Metabolic Disease*.

[14] Cao Y., Liu W., Hui L. (2016). Renal tubular injury induced by ischemia promotes the formation of calcium oxalate crystals in rats with hyperoxaluria. *Urolithiasis*.

[15] Roodnat J. I., de Mik-van Egmond A. M., Visser W. J. (2017). A successful approach to kidney transplantation in patients with enteric (secondary) hyperoxaluria. *Transplantation Direct*.

[16] Hoppe B., Niaudet P., Salomon R. (2017). A randomised phase I/II trial to evaluate the efficacy and safety of orally administered oxalobacter formigenes to treat primary hyperoxaluria. *Pediatric Nephrology*.

[17] Lieske J. C. (2017). Probiotics for prevention of urinary stones. *Annals of Translational Medicine*.

[18] Langman C. B., Grujic D., Pease R. M. (2016). A double-blind, placebo controlled, randomized phase 1 cross-over study with ALLN-177, an orally administered oxalate degrading enzyme. *American Journal of Nephrology*.

[19] ClinicalTrials.gov Bethesda (MD): national library of medicine (US). 2000 Feb 29 -. Identifier NCT03456830. Evaluate ALLN-177 in patients with enteric hyperoxaluria. NCT03456830.

[20] Ermer T., Kopp C., Asplin J. R. (2017). Impact of regular or extended hemodialysis and hemodialfiltration on plasma oxalate concentrations in patients with end-stage renal disease. *Kidney International Reports*.

[21] Tang X., Voskoboev N. V., Wannarka S. L., Olson J. B., Milliner D. S., Lieske J. C. (2014). Oxalate quantification in hemodialysate to assess dialysis adequacy for primary hyperoxaluria. *American Journal of Nephrology*.

[22] Elgstoen K. B. P., Johnsen L. F., Woldseth B., Morkrid L., Hartmann A. (2010). Plasma oxalate following kidney transplantation in patients without primary hyperoxaluria. *Nephrology Dialysis Transplantation *.

